# Multiple factors on driving load in mountain area at night based on factor analysis

**DOI:** 10.1371/journal.pone.0315180

**Published:** 2024-12-06

**Authors:** Hao Li, Heng Jiang, Jiabao Yang

**Affiliations:** 1 School of Civil Engineering Architecture and the Environment, Hubei University of Technology, Wuhan, China; 2 Key Laboratory of Health Intelligent Perception and Ecological Restoration of River and Lake, Ministry of Education, Hubei University of Technology, Wuhan, China; University of Lagos Faculty of Engineering, NIGERIA

## Abstract

Different driving environments may lead to increased mental workload and fatigue among drivers, consequently diminishing driving safety. To investigate the impact of various factors on drivers’ driving load, this study approaches the issue from three perspectives: external weather environment, road driving environment, and in-vehicle driving environment. Through the experimental modeling of the mountainous road section of Provincial Highway S208 in Chongyang County, Xianning City, and employing simulated driving experiments, various factor combinations were designed to investigate the visual characteristics of drivers. Through factor analysis, a comprehensive examination is conducted on four visual indicators: the rate of change in pupil area, fixation duration, saccade velocity, and saccade angle, to explore the patterns of influence exerted by these factors on the driving load of drivers. The evaluation results of driving workload indicate that the degree of driver distraction decreases when the plant spacing is set at 6 meters and the type of road traffic auxiliary facilities is configured to two. When the traffic flow on the road is zero and no driving sub-mission are present, the driver experiences the minimum level of workload. The findings of this study provide robust theoretical support for nighttime mountain driving safety, contributing to the in-depth exploration of traffic safety theories.

## 1. Introduction

The National Bureau of Statistics [1] indicated that, as of the end of 2022, the total number of road traffic accidents in China was approximately 235,000. The number of casualties from road traffic accidents was about 58,000. Although this figure has decreased compared to previous years, it remains significantly high. Numerous adverse interference factors influence driving behavior, which can be categorized into three primary sources: unfavorable external climatic conditions, road driving environments, and in-vehicle driving environments. These adverse factors exert direct or indirect impacts on drivers’ behavior, psychological, and physiological states, thereby affecting their driving skills, inducing negative emotions, diminishing their information perception and emergency response capabilities, ultimately undermining the reliability of driving behavior and the safety of driving. If we start by examining the relationship between various factors and traffic safety, and investigate the impact of these factors on driver workload, it is anticipated that the incidence of traffic accidents can be reduced.

With the continuous advancement of simulation technology, the academic community has conducted in-depth research on the relationship between road driving environments and driving safety. Guo [2] posits that VR safety training yields superior efficacy in enhancing individuals’ risk identification capabilities. Fu [3] came to the conclusion that Visual cues can quickly and accurately improve the ability to identify hazards. Wang et al [4] discovered that the integration of linear visual guidance facilities significantly enhanced driving safety. Han [5] posits that when a driver’s visual features are at the S3 TSIV level, the driver experiences the lowest psychological stress and reduced driving workload. He [6] studied and elaborated the indicated significant variations in the impact of the sidewall effect on driver attention distribution and vehicle stability across different lanes. Drivers in the left lane were the most affected and had the highest driving risk, followed by those in the right lane; those in the middle lane had the least driving risk. Li and Zhang [7] conducted a fine-grained analysis of landscape factors using UC-win/road, suggesting that appropriate vegetation density can effectively guide drivers on curved slopes, maintaining a pleasant emotional state among drivers. Yin et al. [8] conducted a study and elucidated the impacts of roadside trees on the traffic environment, particularly in terms of potential safety hazards such as obscuring traffic signs. Zhu et al. [9] conducted simulation experiments to compare and found that linear guidance signs more effectively display road alignment, capture drivers’ attention, and maintain drivers in a favorable psychological state while navigating through sections. Fang’s [10] research indicates that the combination of multiple types of facilities can provide multi-level linear guidance and contour guidance functions, which are conducive to road traffic safety. Wei et al. [11] pointed out that variations in traffic volume have a considerable impact on drivers’ mental workload. Dibben N et al. [12] revealed in a survey of 1,780 adult drivers that nearly 70% of them admitted to engaging in auditory secondary tasks (such as making phone calls, listening to music, or tuning into radio broadcasts) during the process of driving.

However, previous studies have predominantly focused on the influence of individual factors on driving behavior, with insufficient exploration of the comprehensive effects of multiple factors. Moreover, in assessing the load imposed on drivers by the existing environment, evaluations of driving stability typically focus on superficial and intuitive indicators, with insufficient emphasis placed on deeper metrics such as drivers’ visual perception. Lastly, previous studies have predominantly focused on visual behaviors and workload during daytime on highways and urban roads, with relatively fewer investigations on nighttime mountain roads. Therefore, the objective of this paper is to investigate the visual load on drivers when navigating mountainous roads at night under the combined influence of various adverse driving factors, thereby identifying safer driving environments.

This study employs the UC-win/Road software, Dikablis Glasses 3 eye tracker, and a driving simulator to quantify various load factors. By conducting controlled variable analysis on visual metrics across different nighttime road scenarios, a quantitative model of driving load is established. Finally, the driving load evaluation values of 10 simulation scenarios are ranked. The conclusions of this study will provide a theoretical basis for safe driving on mountainous roads at night.

## 2. Materials and methods

This study employs the UC-win/Road software for the modeling of roadways. By utilizing the Dikablis Glasses 3 eye-tracking device, drivers can simulate experimental procedures during actual driving, thereby obtaining visual representation data. Additionally, Excel was utilized to comprehensively organize the collected data based on the Lyddane-Shindo method, and further analysis and interpretation of driving workload were conducted through factor analysis. Subsequently, the aforementioned data were screened and processed using SPSS software. Finally, a comparative and in-depth analysis and evaluation were conducted on the data obtained from the experiment.

### 2.1 Experimental instrument

In this evaluation, the Dikablis eye tracker, designed by Ergoneers GmbH, was employed. The resolution of the pupil tracking camera is 384 pixels by 288 pixels, with an accuracy of 0.05°and a sampling rate of 60 Hz. Throughout the evaluation process, the data acquired by the eye tracker should be utilized in conjunction with its accompanying software, D-Iab, in the ".txt" format. Save the file format and subsequently export it to Excel for post-test processing and analysis.

### 2.2 Visual behavior characteristic index

GAVAS R [13] and Duan [14] have concluded that changes in pupil area not only reflect human visual adaptation to changes in illumination but can also be used to characterize the magnitude of mental workload and the degree of psychological stress. Therefore, the rate of change in the pupil area, R_t_, can accurately measure the level of driving load experienced by the driver. Shen [15] posits that an extended duration of gaze time, T_d_, correlates with heightened visual load experienced during tunnel transit. Saccades average speed recorded as V_S_. Chen [16] introduced the dynamic variation of sweep angle, denoted as ΔS_a_, which represents the absolute difference between two adjacent sweep angles. This metric signifies the change in adjacent scanning angles and reflects the stability of the driver’s gaze during the driving process.

This study selects parameters that can be accurately measured in experiments, and calculates four visual indicators that reflect the driver’s load state. Detailed information on the visual feature indicators is presented in Table 1.

**Table 1:** Visual feature index.

Index number	Index	Unit
A	Change Rate of Pupil Area R_t_	%
B	Fixation Duration T_d_	s
C	Saccades Velocity V_S_	°/s
D	Dynamic Change of Saccades Angle ΔS_a_	°

### 2.3 Driver selection

According to the data provided by the Transportation Bureau [17], the ratio of male to female drivers is approximately 7:3. A total of 20 test drivers were recruited, comprising 14 males and 6 females. The age of the drivers ranged from 21 to 50 years, with a mean age of 30.1 years and a standard deviation of±10.5 years. The average driving age is 7.4 years, with a standard deviation of±6.2 years. All participants held valid driver’s licenses, possessed certain driving experience, were capable of independently completing driving tasks, enjoyed good physical health, had adequate rest prior to the examination, and had normal or corrected vision of 1.0 or above for both eyes. Table 2 presents the basic information of the drivers.

**Table 2:** Driver’s condition.

Gender	Age			Years of driving		
	Under 24 years of age	24–40 years old	Over 40 years old	3 to 5 years	5 to 10 years	Over 10 years
Male	4	7	3	7	4	3
Female	2	2	2	3	2	1
Total	6	9	5	10	6	4
	20			20		

### 2.4 Experimental path selection

**Fig 1:**
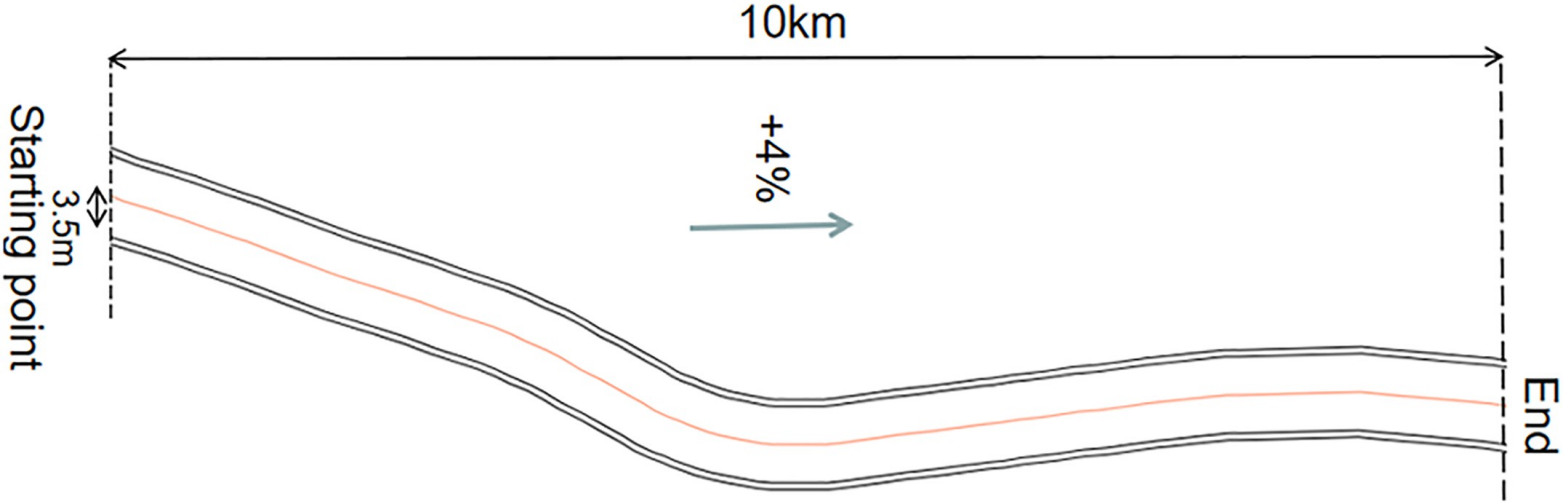
Experimental road alignment and landscape layout.

Based on the research characteristics and practical requirements of this study, in order to gain a deeper understanding of the impact of mountainous road test configurations and traffic flow on drivers’ driving behaviors, a representative secondary road in Hubei Province was selected for investigation and statistical analysis. After thorough consideration of factors such as geographical location, curve profile, weather conditions, and experimental safety, the S208 section in Chongyang County, Xianning City, was ultimately selected as the subject of investigation, spanning a total length of 50.8 kilometers. A 10 km section was selected as the reference scenario for the simulation model, featuring a gradient of +4%. The experimental section consists of a bidirectional, two-lane road with each lane measuring 3.5 meters in width, as illustrated in Fig 1.

## 3. Results

### 3.1 Study on influencing factors of mountain road at night

#### 3.1.1 Plant spacing factor

**Fig 2:**
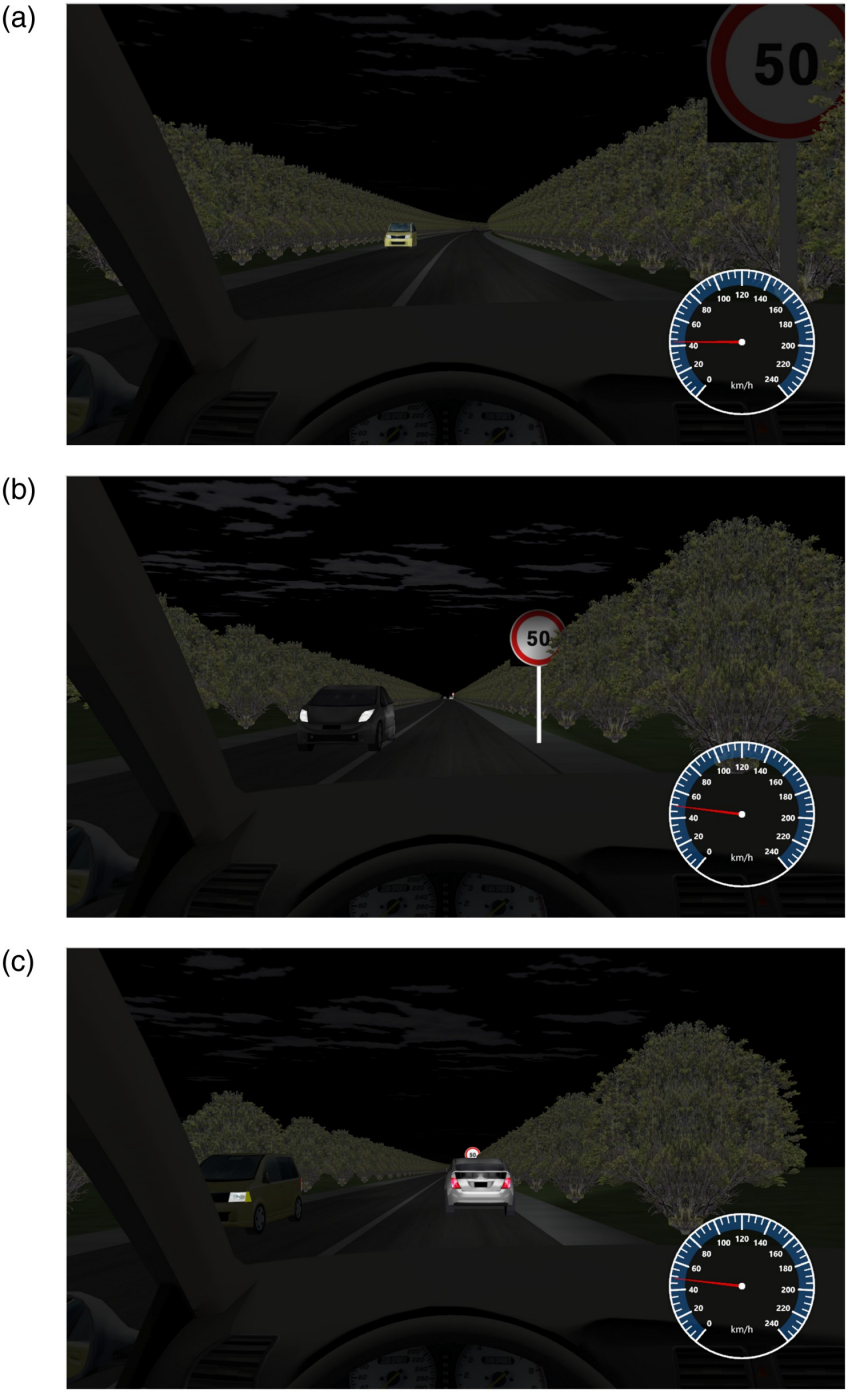
Spacing plant landscape. (a)3m; (b)6m; (c)9m.

Based on the actual field conditions, the spacing is planned at 3m, 6m, and 9m. Within the three levels of plant spacing factors, R_t_ varies with the movement of the vehicle. Table 3 presents the analysis results under the plant spacing factor. The simulation experiment scenario is illustrated in Fig 2.

**Table 3:** Index analysis results under different spacing levels.

Visual behavior index	Plant spacing level		
	Level 1: 3m	Level 2: 6m	Level 3: 9m
Mean R_t_ /(%)	13.3	12.3	12.4
Mean ΔS_a_ /(°)	4.366	4.121	4.144

Among them, in conjunction with the driver’s driving experience, the R_t_ corresponding to Level 1 is slightly larger. This may be due to the fact that scenes with small vegetation spacing require drivers to devote excessive attention and reaction speed when navigating narrow roads, thereby generating additional driving stress. In contrast, within the scenarios corresponding to Levels 2 and 3, the roadside vegetation is appropriately spaced, creating an open and pleasant view, which gives the impression of greater driving space, resulting in a correspondingly smaller R_t_. However, Li [18] posits that when the distance is regulated at 16 meters, the lighting and shadow effects are diminished, resulting in less sensitivity to visual stimuli for the driver, which significantly increases the driver’s cognitive load.

#### 3.1.2 Road traffic facilities factor

**Fig 3:**
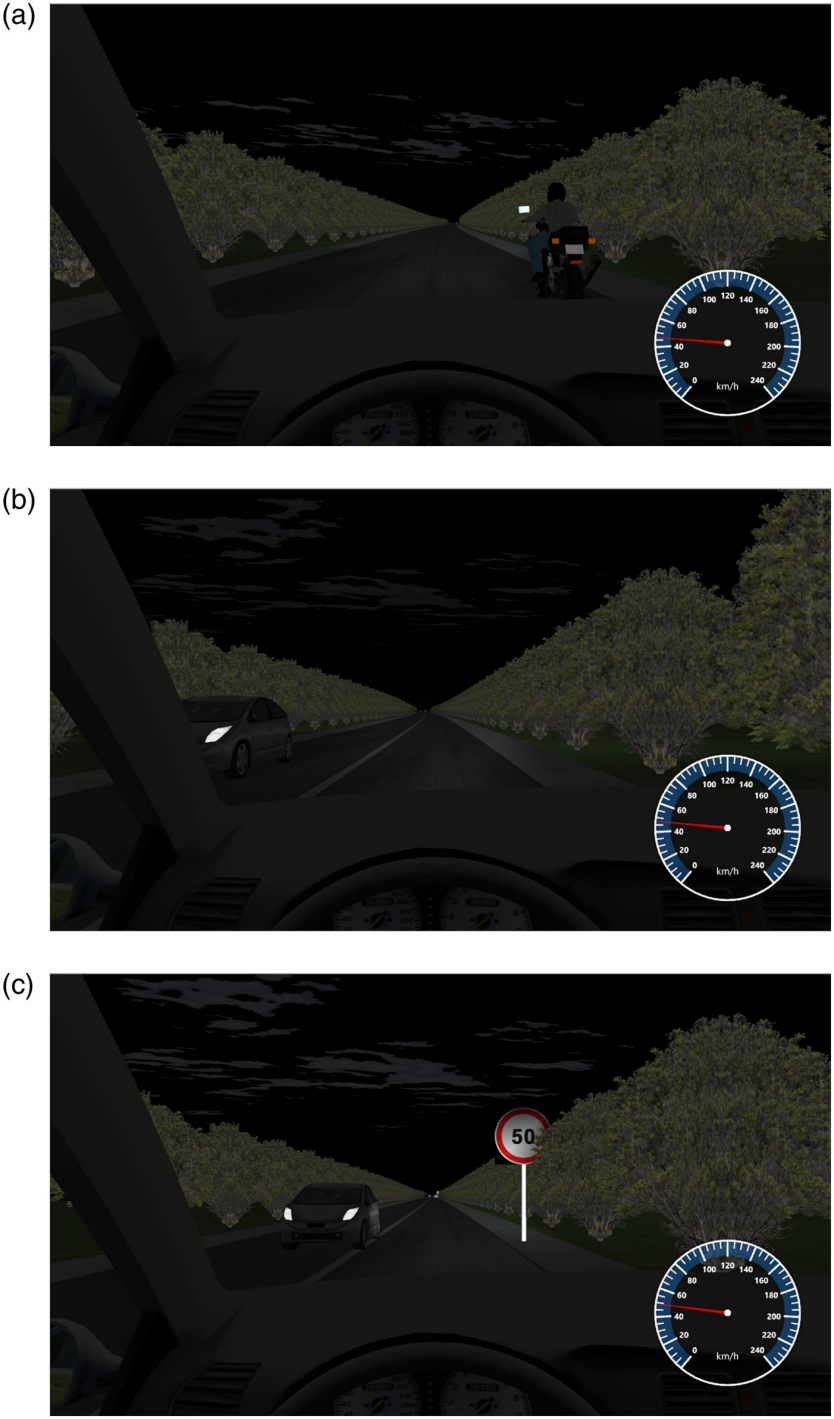
Traffic sign. (a)0; (b)1; (c)2.

In this study, the types of traffic auxiliary facilities on mountainous roads are categorized as 0, 1, and 2. The first scenario indicates the absence of regulatory markings and lines. The second scenario illustrates a setup exclusively featuring lane lines or markers. The third scenario involves the configuration of traffic signs and markings being fully compliant with the regulations and clearly discernible. The study selected three scenarios of traffic ancillary facilities, namely 0, 1, and 2, as the research subjects to further investigate the visual changes induced in participants after driving a vehicle. Table 4 shows the analysis results under the influence of traffic facilities. The simulation experiment scene is shown in Fig 3.

**Table 4:** Index analysis results under different traffic facility number levels.

Visual behavior index	Traffic ancillary facilities		
	Level 1: 0	Level 2: 1	Level 3: 2
Mean R_t_/(%)	16.2	14.0	12.4
Mean ΔS_a_/(°)	3.571	3.941	4.144

By analyzing the actual operational perception of drivers, it is observed that in the nocturnal mountainous environment, when linear guidance is not provided to delineate the linearity of the road, drivers are unable to anticipate upcoming road conditions, leading to increased psychological stress and the need for heightened attentional focus, this is consistent with Wu’s [19] findings, this also explains the phenomenon that the R_t_ at level 1 is higher than that at other levels. The R_t_ at Level 2 is significantly higher than that at Level 3, which may primarily be due to the absence of reflective signs conforming to standards and road lighting along the route, making the direction of the road more ambiguous to drivers at night. The road alignment at Level 3 is clear, with traffic facilities providing advance indication of the upcoming route, ensuring drivers enjoy a favorable driving experience and are subject to appropriate constraints.

#### 3.1.3 Traffic volume factor

**Fig 4:**
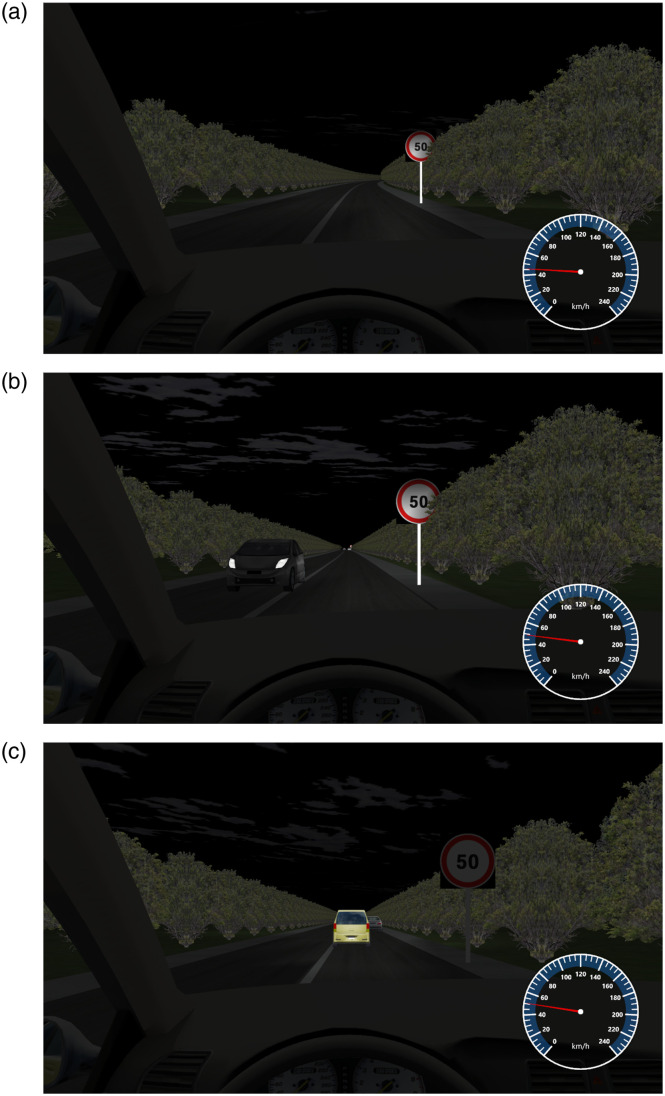
The volume of traffic. (a)0; (b)1; (c)2.

On the evening of January 13, 2024, the S208 section of Chongyang County, Xianning City, has a small traffic flow. Through the fixed observation station method (manual measurement), the number of vehicles passing per hour was recorded as 58. To better simulate the experiment, the traffic flow approximates one vehicle per minute. On this basis, the traffic flow is divided into three distinct levels within the scenario model, namely 0 vehicles per minute, 1 vehicle per minute, and 2 vehicles per minute. Table 5 shows the results of the analysis of visual behavior indicators under the traffic volume factor. The simulation experiment scene is shown in Fig 4.

**Table 5:** Index analysis results under different traffic volume levels.

Visual behavior index	Traffic volume		
	Level 1: 0vpm	Level 2: 1vpm	Level 3: 2vpm
Mean R_t_/(%)	10.2	12.4	16.9
Mean ΔS_a_/(°)	7.416	4.144	3.494

Among them, the ΔS_a_ at level 3 is significantly lower than at other levels, which may be due to the relatively narrow roads in nighttime environments. Wei [11] posits that when encountering or being overtaken, drivers face heightened stress, necessitating intense focus on the road ahead, thereby requiring a more cautious approach. In contrast, the ΔS_a_ at level 1 is higher, which means that when the traffic volume is small or even zero, the driver is more relaxed and the driving load is smaller.

#### 3.1.4 Driving sub-mission factors

In this study, the driving sub-mission is divided into three scenarios. Scenario 0 involves focusing solely on driving, without engaging in activities such as making phone calls or listening to the radio. Scenario 1 for listening to local radio stations while driving. Scenario 2 are hands-free while driving hands-free call while driving (the driver hears and answers a two-digit addition and subtraction question every 5 seconds). Table 6 shows the results of the analysis of visual behavior indicators under the driving sub-mission.

**Table 6:** Index analysis results under different driving sub-mission levels.

Visual behavior index	Driving sub-mission		
	0	1	2
Mean R_t_/(%)	12.4	15.3	20.0
Mean ΔS_a_/(°)	4.144	3.181	2.868

As evidenced by the table, the maximum value of ΔS_a_ is observed at Level 1, indicating that the driver experiences the lowest level of workload in the absence of driving tasks. The execution of driving submission tasks will to some extent occupy the auditory and cognitive resources of the driver, resulting in a decrease in ΔS_a_, indicating an increase in driving load. The third level imposes a greater load than the second level, as it requires analysis and comprehension upon hearing the question. Nurullah [20] has validated this outcome, and the processing of information while listening to the radio is relatively straightforward, primarily involving the reception and understanding of linguistic information.

### 3.2 The influence of multi-factor combination on driving load at night

The optimal combination is characterized by a plant spacing of 6 meters, two types of traffic signs, a traffic volume of zero, and no driving sub-mission. This paper constructs a standard scenario (Scenario 1) based on the aforementioned context. Scenes 2 through Scene 9 constitute a set of 12 scenarios constructed within a driving simulator. After the elimination of duplicate scenes, 8 scenes remain. The 10th scenario is the one with the smallest ΔS_a_ based on a single control factor: the distance between plants is 3m, there are no signs and lines, the traffic volume is 2 vehicles /min, and it also includes the configuration combination of multiple factors of mobile phone (hands-free) driving project. The results of visual data standardization are shown in Table 7.

**Table 7:** Standardized processing results.

Results of standardized processing				
Scene	ZX_1_	ZX_2_	ZX_3_	ZX_4_
1	-1.759	-4.427	-1.678	-1.872
2	-1.278	-0.875	-1.331	-1.029
3	-1.081	-1.116	-1.248	-0.919
4	-1.300	-0.850	-1.331	-1.042
5	-0.447	-0.253	-0.502	-0.416
6	-0.928	-0.655	-1.206	-0.779
7	-0.293	-0.170	-0.294	-0.270
8	-1.212	-0.916	-1.289	-1.016
9	0.385	0.510	0.617	0.308
10	2.814	1.574	1.633	2.448

#### 3.2.1 Quantitative model of driving load

**Fig 5:**
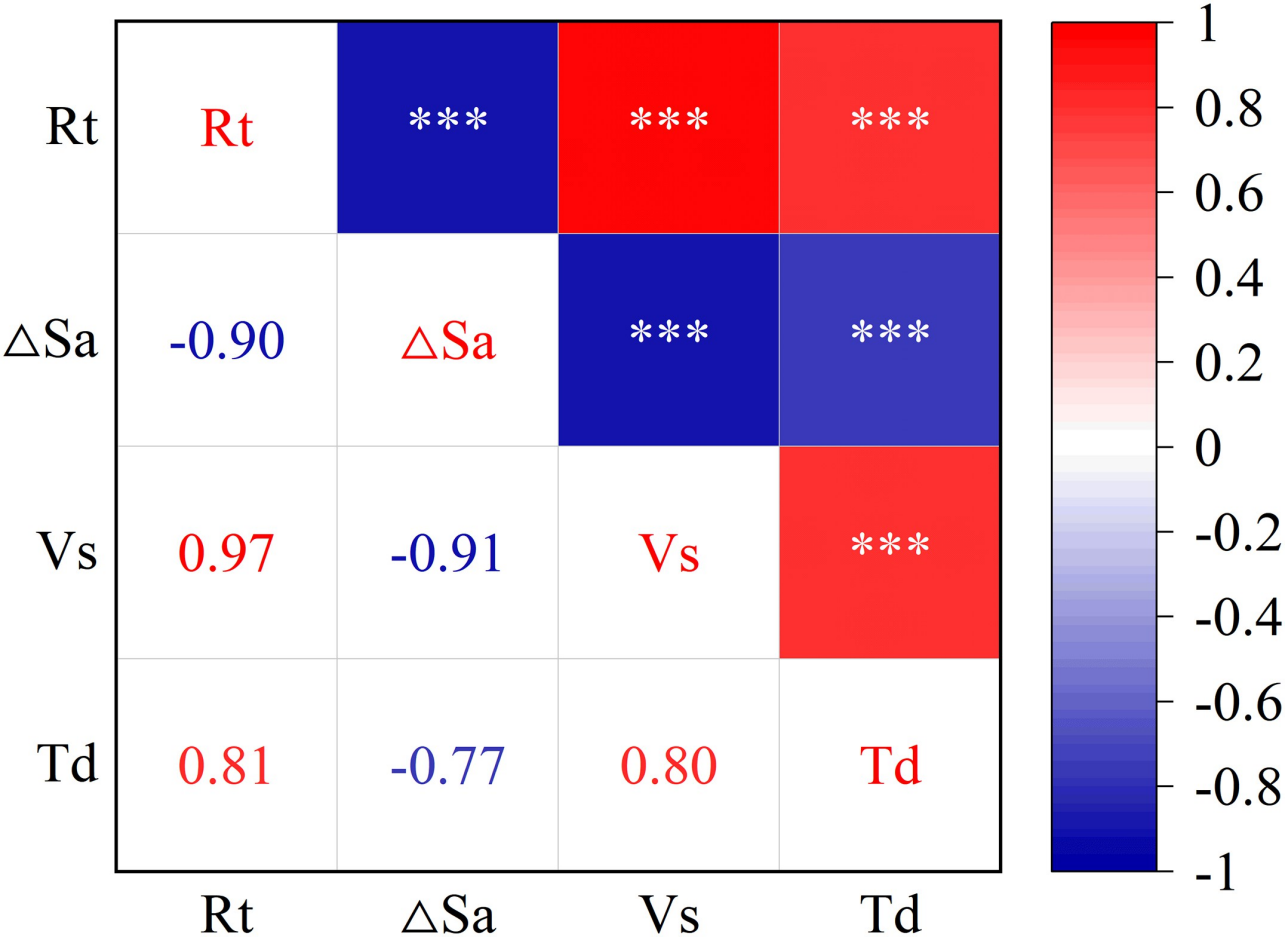
Correlation between visual indicators. * p<=0.05 ** p<=0.01 *** p<=0.001.

Factor analysis can reduce dimensionality when screening and extracting latent relationships among multiple indicators. However, in cases with fewer indicators, comprehensive evaluation can be conducted if the model results are reasonable. This paper selects four visual indicators: Rate of pupil area change Rt, Fixation Duration T_d_, Saccades Speed V_S_, Saccades Angle Dynamic of Change ΔS_a_. Due to the interrelated and interdependent nature of these indicators, which reflect visual changes to varying degrees, factor analysis was employed to analyze and process the data, thereby deriving a new comprehensive index to represent driving load, quantifying the driving load value of drivers on simulated mountainous nighttime routes. The correlations between visual indicators are shown in Fig 5.

The results show that R_t_, V_S_ and T_d_ increase while ΔS_a_ decreases when driving load increases. Therefore, R_t_, V_S_ and T_d_ have a positive effect on driving load, while ΔS_a_ has a negative effect. Therefore, the inverse formula (NMMS) is used to turn the negative action of ΔS_a_ forward, that is, ΔS_a_ uses inverse index in factor analysis, which is represented by ΔS_a1_ in this paper. The Z-score method is used to standardize the data conversion before the factor analysis process can be carried out.

KMO and Bartelett sphericity test were used to determine whether the data were suitable for factor analysis. As can be seen from Table 8, the measured value of KMO sampling moderation is 0.845>0.7, and Sig.<0.01, indicating that there is a high correlation among indicators, the correlation coefficient matrix is a non-unit matrix, factor analysis is effective, and common factors can be extracted from the original variables. KMO and bartlett sphericity tests are shown in Table 8.

**Table 8:** KMO and Bartelett sphericity test.

KMO sample appropriateness measure		0.845
	Approximate chi-square value	438.450
Bartelett sphericity test	Dof	6
	Sig.	0.000

The Z-score method is used to standardize the data conversion before the factor analysis process can be carried out. The common method of extracting common factors is principal component analysis, and the main difference between principal component analysis and factor analysis is whether the factors are rotated.

The higher the degree of commonality, the higher the explanation degree of factor analysis to the original variable, and the rationality of the selected index is also explained. As can be seen from Table 9, the common degree (common variance) of these four variables is greater than or equal to 0.900, which also indicates that the extracted common factor (principal component) can well reflect the main information of the original variable, and the special factor can be ignored. Common degree of variables are shown in Table 9.

**Table 9:** Common degree of variables.

Standardized variable	Initial	Extract
Z-score(R_t_)	1.000	0.960
Z-score(T_d_)	1.000	0.999
Z-score(V_S_)	1.000	0.967
Z-score(ΔS_a1_)	1.000	0.928

Although visually only four indicators are present, the variance contribution rates of the first common factor before and after factor rotation are 89.677% and 61.378%, respectively. This also indicates that, although the number of indicators is limited, it is reasonable to utilize factor analysis to extract the intrinsic relationships within the original data. As can be seen from Table 10, if the factor is not rotated, the first common factor (principal component) should be selected, whose variance contribution rate is 89.677% and corresponding eigenvalue is 3.587. The eigenvalue of the second principal component is 0.267<1, which does not meet the principal component selection criteria. Orthogonal rotation of factors using the Maximum Variance Method was employed, with the first two common factors being preferentially selected. Both of these factors have eigenvalues greater than 1, and their cumulative variance contribution rate reaches 96.356%. The specific selection of the first principal component before rotation (principal component analysis) or the first two common factors after rotation should be determined based on whether the relationship between common factors and indicators in the load matrix can reasonably explain the relationship between driving load and visual indicators. Total variance explanation are shown in Table 10.

**Table 10:** Total variance explanation.

Element	Initial eigenvalue			Extract the sum of squared loads			Rotating load sum of squares		
	total	variance percentage	grand total%	total	variance percentage	grand total%	total	variance percentage	grand total%
1	3.587	89.677	89.677	3.587	89.677	89.677	2.455	61.378	61.378
2	0.267	6.679	96.356	0.267	6.679	96.356	1.399	34.978	96.356
3	0.113	2.815	99.171						
4	0.033	0.829	100.00						

The factor load matrix before rotation is also the principal component load matrix, reflecting the degree of correlation between the common factor (principal component) and the original variable. As can be seen from Table 11, in the first principal component, the load of R_t_, T_d_, V_S_ and ΔS_a1_ reaches 0.973, 0.890, 0.973, and 0.948 respectively, that is, the four indicators are all positive indicators when quantifying driving load. The principal component can reasonably explain the driving load, and the contribution rate of the four indexes is R_t_ = V_S_>ΔS_a1_>T_d._ Unfactored analysis component matrix are shown in Table 11.

**Table 11:** Unfactored analysis component matrix.

Standardized variable	Common factor (principal component 1)
Z-score(R_t_)	0.973
Z-score(T_d_)	0.890
Z-score(V_S_)	0.973
Z-score(ΔS_a1_)	0.948

The expression of the principal component score is:

$$\text{F}=\frac{1}{\sqrt{3.587}}(0.973Z_{1}+0.890Z_{2}+0.973Z_{3}+0.948Z_{4})$$ (1)

Where Z_1_, Z_2_, Z_3_and Z_4_ respectively represent standardized original variables. The factor model is:

$${\text{Z}}_{1}=0.973{\text{F}}_{1}$$ (2)

$${\text{Z}}_{2}=0.890{\text{F}}_{1}$$ (3)

$${\text{Z}}_{3}=0.973{\text{F}}_{1}$$ (4)

$${\text{Z}}_{4}=0.948{\text{F}}_{1}$$ (5)

During factor analysis, it is customary to perform rotational transformations on the factor loading matrix to better elucidate the practical significance of the common factors. Table 12 presents the rotated factor loading matrix, from which it is evident that the first two common factors are directly proportional to the four indicators. In the first common factor, the load of R_t_, V_S_ and ΔS_a1_ reaches 0.86, 0.871 and 0.856 respectively, while the load of T_d_ is only 0.457. However, in the second common factor, the T_d_ load reaches 0.889, while the other three indicators load are 0.476, 0.456 and 0.476, respectively. Combined with practical significance, it can be seen that the first common factor represents the driving load of three key quantitative indicators other than fixation time. The second common factor represents the driving load with fixation time as the key quantitative index. According to the variance explanation table, the contribution variance of the two common factors reached 96.356%, while the contribution rate of the first common factor (principal component) before rotation was 89.677%, which was smaller than that after rotation. Moreover, the factor load array after rotation represented the driving load more comprehensively. Therefore, the first two common factors after rotation are selected as the calculation basis of the quantization model. This also shows that factor analysis is more suitable for the experimental data in this paper than principal component analysis when constructing evaluation model. Factor analysis component matrix are shown in Table 12.

**Table 12:** Factor analysis component matrix.

Standardized variable	Common factor 1	Common factor 2
Z-score(R_t_)	0.856	0.476
Z-score(T_d_)	0.457	0.889
Z-score(V_S_)	0.871	0.456
Z-score(ΔS_a1_)	0.856	0.476

The factor model after rotation is:

$${\text{Z}}_{1}=0.\;856{{\text{F}}_{1}}^{’}+0.\;476{{\text{F}}_{2}}^{’}$$ (6)

$${\text{Z}}_{2}=0.\;457{{\text{F}}_{1}}^{’}+0.\;889{{\text{F}}_{2}}^{’}$$ (7)

$${\text{Z}}_{3}=0.\;871{{\text{F}}_{1}}^{’}+0.\;456{{\text{F}}_{2}}^{’}$$ (8)

$${\text{Z}}_{4}=0.\;856{{\text{F}}_{1}}^{’}+0.\;476{{\text{F}}_{2}}^{’}$$ (9)

Where F_1_ ’and F_2_’ are the common factor scores after rotation respectively.

The factor score coefficient matrix was obtained by principal component analysis, as shown in Table 13. Negative numbers occur relative to the average and are caused by the transfer effect of data processing and do not affect the calculation results. Factor score coefficient matrix are shown in Table 13.

**Table 13:** Factor score coefficient matrix.

Standardized variable	Common factor 1	Common factor 2
Z-score(R_t_)	0.468	-0.186
Z-score(T_d_)	-0.792	1.527
Z-score(V_S_)	0.523	-0.263
Z-score(ΔS_a1_)	0.582	-0.357

The scoring function of two common factors can be written as:

$${{\text{F}}_{1}}^{’}=0.468Z_{1}-0.792Z_{2}+0.523Z_{3}+0.582Z_{4}$$ (10)

$${{\text{F}}_{2}}^{’}=-0.186Z_{1}+1.527Z_{2}-0.263Z_{3}-0.357Z_{4}$$ (11)

Calculate the score of the original variable on two common factors (F_1_’, F_2_’). Taking the variance contribution rate corresponding to each common factor as the weight, and taking the sum of variance contribution rate as the basis for weight unitization, combined with the comprehensive linear weighting model, the calculation formula of the overall driving load quantization index S is finally obtained as follows:

$$\text{S}=\frac{1}{0.96356}(0.61378{{\text{F}}_{1}}^{’}+0.34978{{\text{F}}_{2}}^{’})$$ (12)

Since the driving load value S calculated by the factor analysis method is positive and negative, all data are converted into the interval of [0,1] by the extreme value method, so as to facilitate subsequent analysis and comparison.


$${\text{S}}_{\text{i}}^{*}=\left\{\begin{array}{c} \frac{{\text{S}}_{\text{i}}-{\text{minS}}_{\text{i}}}{{\text{R}}_{\text{i}}}\\ {\text{R}}_{\text{i}}\neq 0\\ 0.5\;{\text{R}}_{\text{i}}=0 \end{array}\right.\text{i}=1,2,\cdots ,\text{x}$$ (13)

Where R_i_ is the max-min.

**Fig 6:**
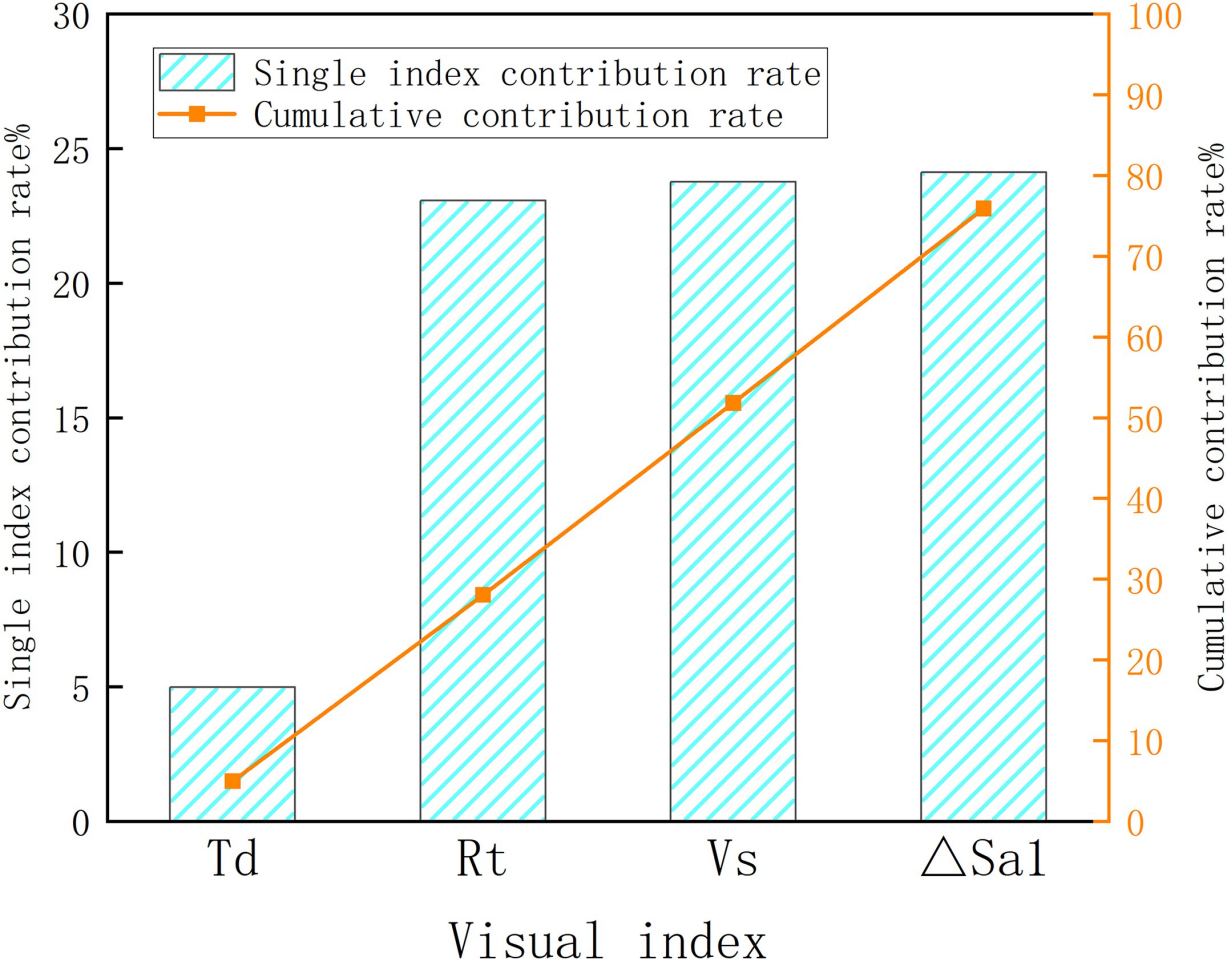
Contribution rate of each index in driving load model.

Multiply the factor score coefficient matrix by the variance contribution rate corresponding to each common factor to get the contribution rate of each index in the final driving load calculation model, as shown in Fig 6. The contributions to the driving load calculation results are as follows: ΔSa_1_>V_S_>R_t_>T_d_ (24.11%, 23.77%, 23.06%, 4.98%). It also shows that visual saccades and changes in pupil area have higher validity and reliability than fixation indicators in calculating driving load.

#### 3.2.2 Analysis of driving load by combination of multiple factors at night

**Fig 7:**
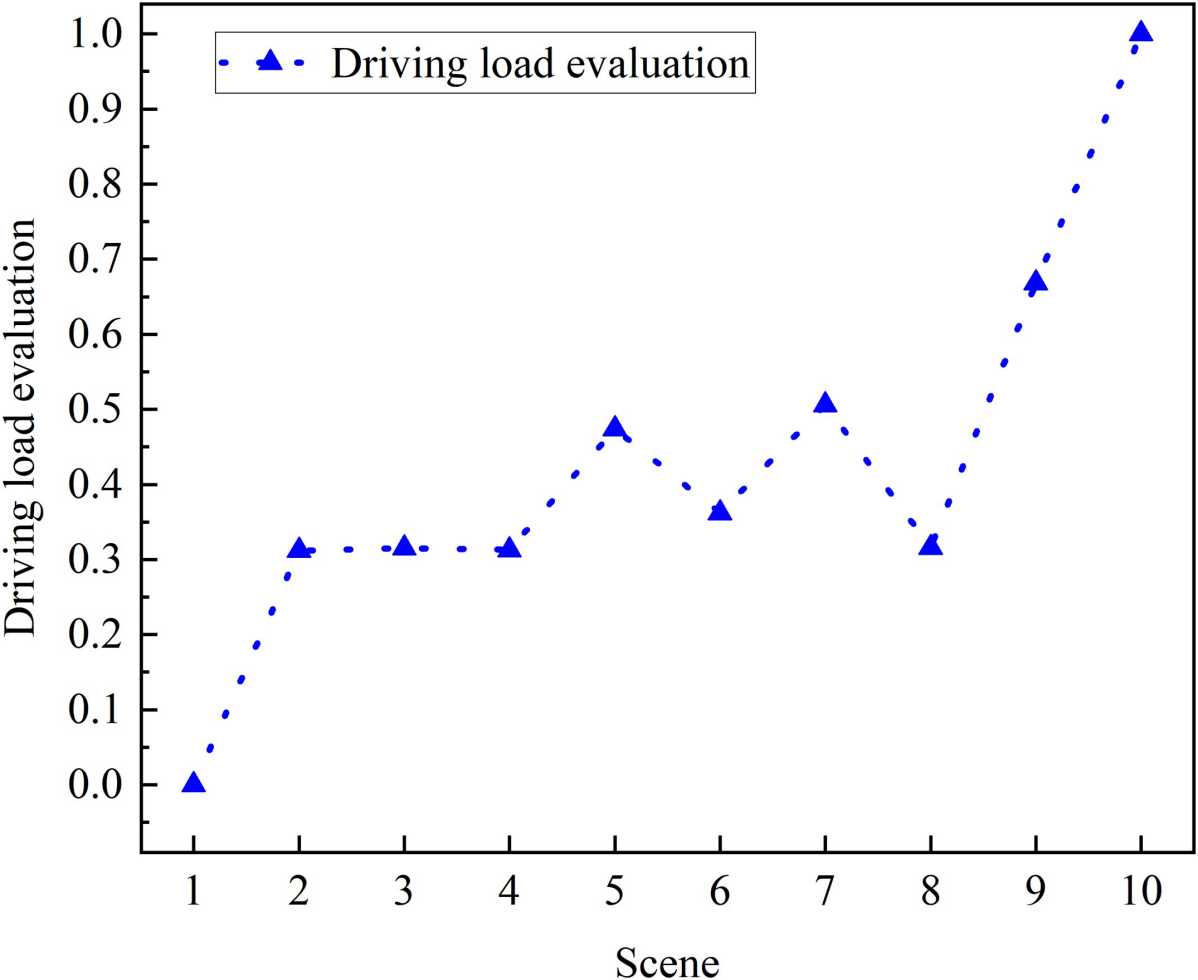
Line chart of stability for each factor at each level.

Table 14 shows the driving load results under various factors and levels. Fig 7 shows the line chart of stability for each factor at each level. According to the comprehensive analysis of Table 14 and Fig 7, scenario 1 has the least load. Scenario 2–4: On the premise of maintaining other factors unchanged, adjust the spacing of plants, the maximum driving load can be achieved when the spacing of plants is set to 3m. Scenarios 1, 5 and 6: While maintaining other factors constant, the alteration of the type of traffic signs reveals an increase in driving load as the number of traffic signs decreases. Scenario 1, 2 and 7: By changing the traffic volume, when the traffic volume is 0, the driving load is minimal, but when the traffic volume is increased to 2, the driving load will be greatly increased. Scenario 2, 8 and 9: The impact of the driving submission was observed, revealing that conversing imposes a significant load on the driver, whereas listening to the radio results in a comparatively lighter load. Scenario 10 represents the combination of four corresponding factors, exhibiting the minimal saccade dynamic changes and achieving the top ranking, thereby substantiating that the factors leading to a large load, when combined, yield an even greater load.

**Table 14:** Driving load results under various factors and levels.

Scene	Plant spacing/m	Traffic signs/classes	Traffic volume/vpm	Driving sub-mission	Driving load evaluation	Driving load ranking
1	6	2	0	No	0.000	10
2	6	2	1	No	0.312	9
3	3	2	1	No	0.315	7
4	9	2	1	No	0.313	8
5	6	0	1	No	0.474	4
6	6	1	1	No	0.362	5
7	6	2	2	No	0.506	3
8	6	2	1	Listen to the radio	0.316	6
9	6	2	1	hands-free calls	0.668	2
10	3	0	2	hands-free calls	1.000	1

By combining the descriptions and analyses of drivers’ driving experiences, it can be concluded that driving workload impacts the following aspects:1. When the distance between plants is excessive, drivers may experience a lack of visual stimulation; conversely, when the spacing is too narrow, drivers may feel constrained by the space, thereby exacerbating fluctuations in negative emotions. 2. The more comprehensive the traffic auxiliary facilities, the better. Their scientific and rational utilization can effectively manage and regulate drivers’ behavior. 3. The greater the vehicle flow on narrow roads at night, the higher the stress experienced by drivers. 4. Speaking occupies more cognitive resources for drivers than listening to the radio, thereby rendering driving more perilous.

## 4. Conclusion

1. By comparing the driving load of drivers under various factors, the results show that the driving load of drivers is the smallest when the plant spacing is 6m, there are 2 kinds of traffic facilities, the traffic volume is 0 and there is no driving sub-mission. Considering the driver’s driving experience, this scenario is conducive to the driver’s physical and mental well-being.
2. The traffic flow design in this study is relatively simplistic, with vehicles encountered at a speed of 50 km/h per minute. The time headway distribution of traffic flow and the influence of high beam on drivers can be deeply considered in the follow-up research; This paper exclusively analyzes the visual load of drivers. Subsequent research could integrate electrocardiograms and electroencephalograms for a more comprehensive and in-depth analysis of integrated load.
